# Use of primaquine and glucose-6-phosphate dehydrogenase deficiency testing: Divergent policies and practices in malaria endemic countries

**DOI:** 10.1371/journal.pntd.0006230

**Published:** 2018-04-19

**Authors:** Judith Recht, Elizabeth A. Ashley, Nicholas J. White

**Affiliations:** 1 Mahidol-Oxford Tropical Medicine Research Unit (MORU), Faculty of Tropical Medicine, Mahidol University, Bangkok, Thailand; 2 Myanmar Oxford Clinical Research Unit, Yangon, Myanmar; 3 Centre for Tropical Medicine and Global Health, Nuffield Department of Medicine, University of Oxford, Oxford, United Kingdom; Johns Hopkins Bloomberg School of Public Health, UNITED STATES

## Abstract

Primaquine is the only available antimalarial drug that kills dormant liver stages of *Plasmodium vivax* and *Plasmodium ovale* malarias and therefore prevents their relapse (‘radical cure’). It is also the only generally available antimalarial that rapidly sterilises mature *P*. *falciparum* gametocytes. Radical cure requires extended courses of primaquine (usually 14 days; total dose 3.5–7 mg/kg), whereas transmissibility reduction in falciparum malaria requires a single dose (formerly 0.75 mg/kg, now a single low dose [SLD] of 0.25 mg/kg is recommended). The main adverse effect of primaquine is dose-dependent haemolysis in glucose 6-phosphate dehydrogenase (G6PD) deficiency, the most common human enzymopathy. X-linked mutations conferring varying degrees of G6PD deficiency are prevalent throughout malaria-endemic regions. Phenotypic screening tests usually detect <30% of normal G6PD activity, identifying nearly all male hemizygotes and female homozygotes and some heterozygotes. Unfortunately, G6PD deficiency screening is usually unavailable at point of care, and, as a consequence, radical cure is greatly underused. Both haemolytic risk (determined by the prevalence and severity of G6PD deficiency polymorphisms) and relapse rates vary, so there has been considerable uncertainty in both policies and practices related to G6PD deficiency testing and use of primaquine for radical cure. Review of available information on the prevalence and severity of G6PD variants together with countries’ policies for the use of primaquine and G6PD deficiency testing confirms a wide range of practices. There remains lack of consensus on the requirement for G6PD deficiency testing before prescribing primaquine radical cure regimens. Despite substantially lower haemolytic risks, implementation of SLD primaquine as a *P*. *falciparum* gametocytocide also varies. In Africa, a few countries have recently adopted SLD primaquine, yet many with areas of low seasonal transmission do not use primaquine as an antimalarial at all. Most countries that recommended the higher 0.75 mg/kg single primaquine dose for falciparum malaria (e.g., most countries in the Americas) have not changed their recommendation. Some vivax malaria–endemic countries where G6PD deficiency testing is generally unavailable have adopted the once-weekly radical cure regimen (0.75 mg/kg/week for 8 weeks), known to be safer in less severe G6PD deficiency variants. There is substantial room for improvement in radical cure policies and practices.

## Background

Glucose 6-phosphate dehydrogenase (G6PD) deficiency is the most prevalent enzyme deficiency in the world. An estimated 400 million people are affected (reviewed in [1–5]). Within the erythrocyte, G6PD is the only source of NADPH, necessary for provision of reduced glutathione and the function of catalase. Both reduced glutathione and catalase provide essential cellular protection from oxidative stresses. G6PD activity decreases exponentially as red cells age, with a normal half-life of about 50 days, but in most genetic variants, G6PD degrades more rapidly, rendering older erythrocytes increasingly vulnerable to oxidant stresses and, thus, haemolysis. Very severe G6PD deficiencies are sporadic and rare and cause chronic nonspherocytic haemolytic anaemia. Less severe deficiencies are polymorphic, and they are very common in most tropical areas. This is attributed to the protection G6PD deficiency provides against the adverse effects of malaria [6–9].

As G6PD deficiency is X-linked, males are hemizygous and either phenotypically normal or deficient (although the degree of deficiency depends on the genotype) (Table 1). Homozygote females are as deficient as the hemizygous males, whereas heterozygous females are mosaics with intermediate levels of deficiency as a result of embryonic random X-chromosome inactivation (lyonisation). Their blood contains a mixture of G6PD normal and G6PD deficient (G6PDd) cells. The proportion in the heterozygous population overall averages 50:50, but some heterozygotes may have a majority of G6PDd erythrocytes. Most qualitative G6PD blood screening tests report enzyme activities >30% as ‘normal’, so heterozygotes testing ‘normal’ could still have up to 70% of their red cells G6PDd—and so be vulnerable to oxidant haemolysis [10].

**Table 1:** G6PD genotypes and phenotypes.

If q is the allele frequency for G6PD deficiency and 1-q is the allele frequency for the wild-type gene, and assuming a Hardy-Weinberg equilibrium, then because G6PD deficiency is X-linked, the prevalence of the G6PD deficient genotype in hemizygous males is q, the prevalence in homozygous females is q^2^, and the prevalence in heterozygote females is 2[q(1-q)]. Thus, the allele frequency equals the proportion of males who are G6PDd, and, although a higher proportion of females carry the mutant allele, only a minority of these will be designated as G6PDd using the usual threshold of <30% of normal enzyme activity. For example, if 10% of men are G6PD-deficient hemizygotes, then 1% of women will be equally deficient homozygotes, and 18% of women will be heterozygotes with variable levels of deficiency (because of lyonisation).

Note: Because G6PD deficiency protects against symptomatic vivax malaria, the prevalence of G6PD deficiency in patients requiring radical treatment with primaquine may be lower than in the general population.

The 8-aminoquinoline primaquine has a unique antimalarial activity spectrum. It has prophylactic, radical curative (*Plasmodium vivax* and *P*. *ovale*), and rapid gametocyte sterilising (*P*. *falciparum*) activities (Table 2). The main concern limiting primaquine use is the risk of dose-dependent acute haemolytic anaemia in G6PDd individuals. Uncertainty over the risks and benefits of primaquine, together with lack of readily available G6PD deficiency testing in malaria-endemic areas, has resulted in a wide and confusing array of policies and practices. The historical background to these recommendations and the current primaquine and G6PD deficiency testing policies in malaria-endemic countries are reviewed briefly here.

**Table 2:** Primaquine for malaria treatment.

Malaria*	Primaquine use	Recommended primaquine dose	Comments
***Plasmodium falciparum***	As a gametocytocide (to block transmission of malaria to mosquitoes)	Single-dose treatment along with ACT	WHO-recommended dose 0.25 mg/kg (previously 0.75 mg/kg) **without a requirement for G6PD deficiency testing** [20]
***P*. *vivax* and *P. ovale***	For radical cure to eliminate dormant hypnozoites and so prevent relapses	0.25 mg/kg daily for 14 days with chloroquine or ACT For G6PDd patients, some countries recommend 0.75 mg/kg once weekly for 8 weeks	Some countries in the Americas recommend shorter regimens of 0.5 mg/kg daily for 7 days. Some give primaquine as directly observed treatment

*Uncomplicated *P*. *knowlesi* malaria, prevalent in some Southeast Asian countries, is usually treated with an ACT without primaquine, as *P*. *knowlesi* does not form hypnozoites and its sexual stages (gametocytes) are cleared by the ACT (reviewed in [54]). **Abbreviation:** ACT, artemisinin combination therapy.

### Historical background to current practices

Much of our knowledge about the haemolytic risks of primaquine derives from a comprehensive series of studies conducted in the United States of America on healthy male African American volunteers (presumably G6PD African A- hemizygotes) between the late 1940s and the early 1960s [11–14]. Haemolysis was shown to be dose dependent, with older red cells haemolysing preferentially. With daily primaquine dosing in G6PD A- subjects, haemoglobin concentrations first fell and then rose again, despite continued drug administration, as young, less G6PDd erythrocytes, which were more resistant to oxidant haemolytic effects, entered the circulation. This was exploited in the 8-week radical cure regimen, in which a single 0.75 mg/kg (adult dose 45 mg) dose is taken once weekly. The safety of this regimen was established only in the G6PDd A- variant, which is generally less severe than most other polymorphic variants.

The widely recommended primaquine radical cure regimen for vivax malaria in G6PD-normal patients (0.25 mg/kg/day for 14 days; adult daily dose 15 mg) was based on experience in the Korean War (1950–1953) with long-incubation *P*. *vivax* [15]. This stood unchallenged for 3 decades and, with very few clinical studies, practices began to diverge. In Southeast Asia and Oceania, where the frequently relapsing ‘Chesson’ phenotype *P*. *vivax* is prevalent, the 0.25 mg/kg/day dose was considered insufficient. Higher doses were recommended, culminating today with 0.5 mg/kg/day for 14 days [16]. In contrast, in India, which harbours a substantial proportion of the world’s vivax malaria, 5-day primaquine courses were associated with relapse rates of approximately 20% (compared with ≥50% rates further east) and were recommended for 4 decades until it was discovered that 20% relapse rates occurred whether or not the 5-day regimen was given [17,18]. India now recommends standard 0.25 mg/kg/day 14-day primaquine courses for radical cure. In South America, 7-day 0.5 mg/kg/day regimens are widely recommended.

The gametocytocidal activity of 8-aminoquinolines in falciparum malaria was observed in the first clinical studies nearly 100 years ago and shown soon afterwards to result in rapid sterilisation of the infection [19]. This potent transmission-blocking potential was exploited in recommendations over the past 50 years to add a single dose of primaquine to falciparum malaria treatment, mainly in low-transmission settings, where symptomatic infections contribute substantially to transmission overall. The original single dose was 0.75 mg/kg (adult dose 45 mg), lowered recently to 0.25 mg/kg (single low dose, SLD), which appears to provide the same reduction in transmissibility [20–22] but obviously less haemolytic risk [23].

### Malaria epidemiology and current practices

In Africa, *P*. *falciparum* predominates, although *P*. *vivax* comprises approximately half the malaria in the Horn of Africa and 10% in Madagascar. In the Americas, with the exception of Haiti, *P*. *vivax* predominates, and in Asia, prevalences of *P*. *vivax* and *P*. *falciparum* are approximately equal, with high rates of mixed infection (Table 3). In India, relapse rates following vivax malaria are approximately 20%, and radical treatment is recommended. In East Asia, *P*. *vivax* relapse rates exceed 50% in vivax malaria, and approximately 30% of patients experience a presumed *P*. *vivax* relapse following falciparum malaria, yet a policy of radical cure has never been considered in acute falciparum malaria. For vivax malaria in the Americas, Asia, and Oceania, radical cure is widely recommended but is often not given because G6PD deficiency testing is unavailable.

Use of primaquine as a *P*. *falciparum* gametocytocide has been confined mainly to areas of low transmission [20]. In high-transmission settings, the transmission reservoir of asymptomatic individuals is considered too large for primaquine to have any impact. As countries plan for elimination, this transmission blocking recommendation is being more actively supported.

### G6PD deficiency and primaquine use

The main factor limiting use of primaquine is concern over dangerous haemolysis in patients with G6PD deficiency. The prevalences of G6PD deficiency in malaria-endemic countries range from very low (e.g., the Democratic Republic of Korea) to over 30% [24]. The most frequent G6PDd variant in Africa (A-) was generally considered ‘mild’, although in some cases haemolysis can be severe [25]. In the Americas, G6PD A- is also the most common variant, whereas in Asia and Oceania there are numerous variants, most of which are more severe than A-. The G6PD Mediterranean variant, prevalent from Southern Europe to Southeast Asia, is at the severe end of the spectrum, and in people with this form of G6PD deficiency, repeated primaquine administration can cause life-threatening haemolysis [26]. Most estimates of G6PD deficiency prevalence are based on enzyme activity studies in males, although molecular genotyping methods for epidemiological assessments have predominated in recent years. Genotyping assessments inform risks for males, but they are less informative for females as the majority of those carrying the G6PDd allele will be heterozygotes (Table 1), and most of these females will test as G6PD normal with the usual screens (detecting <30% of normal activity) but may still be at risk of significant haemolysis [9].

## Methods

A review of national policies on primaquine use and recommendations for G6PD deficiency testing is presented with a summary of the prevalences of G6PD deficiency in malaria-endemic countries. This is divided into 3 geographic sections: (a) Africa; (b) Asia, Oceania, and the Middle East; and (c) the Americas. Estimates of G6PD deficiency prevalence were obtained from published reviews and studies, including predictive models based on published data. Current policies for G6PD deficiency testing and primaquine treatment in each country were obtained mainly from the World Health Organization (WHO) World Malaria Report 2016 [27] and updated for 2017 [28] and/or publications from each country, and maps were generated based on these reported policies. In most cases, we confirmed WHO-reported current policies and practices in countries by contacting countries’ ministries of health (MoHs) or researchers and/or institutions in malaria-endemic areas.

## Results

Estimates of G6PD deficiency prevalence and predominant variants are summarised in Tables 4 to 6. These proportions usually refer to the allele frequency, which is equivalent to the proportion of males expected to be G6PDd.

**Table 3:** Malaria, G6PD deficiency, and indications for primaquine in different malaria-endemic regions.

Region	Parasite species	Common G6PDd variants	Recommended primaquine regimens
**Africa**	Mostly *Pf*	Mostly A-	Gametocytocidal single dose for *Pf* in low-transmission areas
**Asia, Oceania, Middle East**	Both *Pf* and *Pv* (close to 50:50 in many countries) *P*. *knowlesi* accounted for 38% of malaria reported cases in Malaysia in 2014 [56]	Several, including Mahidol, Viangchan, Kaiping, Canton, Orissa, and Mediterranean. Generally more severe than A-.	Gametocytocidal single dose for *Pf*; radical cure for *Pv*
**The Americas**	Both *Pf* and *Pv* (overall around 75% *Pv*, 25% *Pf*)	Mostly A-	Gametocytocidal single dose for *Pf*; radical cure for *Pv*

**Abbreviations:** *Pf*, *Plasmodium falciparum*; *Pv*, *P*. *vivax*

### Africa

All malaria-endemic countries in Africa have high estimated G6PD deficiency prevalences, including 7 with >13%–17% and 8 with >20%–23% (Table 4) [29].

**Table 4:** G6PD deficiency prevalences*, testing, and primaquine policies in African malaria-endemic countries.

Country	Estimated G6PD deficiency prevalences for common variants*	G6PD deficiency testing before primaquine**	Single-dose primaquine for Pf**	Primaquine use for Pv**
Bioko Island, Equatorial Guinea	4,144 subjects (66.6% males) by FST showed 8.7% G6PD deficiency [57]; genotyping showed 99.2%, (356/359) A- variant (G202A/A376G)	Required, not implemented	Not used	Not used
Mali, Cameroon°, Guinea, Gabon	>10%–13% [24]	Not required	Not used	Not used
Togo, Benin	>20%–23% [24]	No policy	Not used	Not used
Ghana	>17%–20% [24]	Not required	Not used	Not used
Côte d’Ivoire, Chad	>13%–17% [24]	Not required	Not used	Not used
Comoros	9.5% [59]	Not required	SLD primaquine is policy	Not used
Mayotte	9.5% [59]	Required	SLD primaquine is policy	PQ used as radical treatment (DOT)
Congo, Democratic Republic of Congo°, Zambia, Malawi	>20%–23% [24]	Not required	Not used	Not used
Burkina Faso, Sierra Leone, Liberia, Burundi, Central African Republic, Guinea-Bissau	>7%–10% [24]	Not required	Not used	Not used
Mauritania	>7%–10% [24]	Required since 2014	Not used	Started PQ policy of 0.25 mg/kg/day for 14 days since 2014
Madagascar°	>20%–23% [24]	Not required	*Started SLD PQ policy in low-transmission (pre-elimination) areas in 2015; Pf PQ for children in remote areas not widely implemented because of dosing difficulties*	0.25 mg/kg/day for 14 days since 2015
Kenya	>10%–13% [24]	Not required	Not used	No policy
Ethiopia°	>1%–3% [24,60] *N* = 555 survey in 2014 in the southwest, no G6PDd A- or Mediterranean samples were found by genotyping [58]	Not required	SLD PQ (0.25 mg/kg) introduced recently	*Use of PQ for Pv radical cure in elimination districts with G6PD deficiency testing is planned (not yet implemented)*
Djibouti	No published estimates	Not required	Not used	Not used
The Gambia, Uganda, United Republic of Tanzania, Zanzibar, Zimbabwe, Senegal, Nigeria	>13%–17% [24]	Not required	Implemented in Zanzibar and Zimbabwe (DOT) only	Not used
Sudan°, South Sudan	>13%–17% [24]	Not required	Not used	0.25 mg/kg/day for 14 days in Sudan since 2005 *although not widely implemented*
Mozambique	>20%–23% [24]	No policy	No policy	No policy
Namibia	No published estimates	Not required	SLD PQ policy as DOT	Radical cure as DOT is policy
Sao Tomé and Principe	10.8% [61]	Required	SLD PQ policy as DOT	Radical cure as DOT is policy
Swaziland	>7%–10% [24]	Not required	Started SLD PQ policy as DOT since 2014	No policy
Botswana	>3%–7% [24]	Not required	Started SLD PQ policy as DOT in 2015	0.25 mg/kg/day for 14 days since 2015
Eritrea	>3%–7% [24]	Not required	SLD PQ policy since 2015 (not implemented)	0.25 mg/kg/day for 14 days since 2002 as DOT
Rwanda, Somalia	>3%–7% [24]	Not required	Not used	No policy
Niger	>3%–7% [24]	No policy	Not used	No policy
Angola	>13%–17% [24]	Required since 2006	Not used	0.25 mg/kg/day for 14 days
South Africa	>3%–7% [24]	Yes	Not used	Uses DOT with PQ for *Pv*, regimen not specified; *PQ is no longer registered in the country and is only available for compassionate use*, *for the few cases of Pv and P*. *ovale (14-day course)*
Algeria	ND	Not required	Single dose	0.25 mg/kg/day for 14 days (with CQ) as DOT
Cabo Verde	From 176 individuals, only G6PD A- was found at a low frequency of slightly under 1% [61]	Required	Single dose (DOT)	There is no *Pv* in the country, but 2015 modified policy indicates PQ for *Pv* (not implemented currently)

**Abbreviations:** *Pv*, *Plasmodium vivax*; *Pf*, *Plasmodium falciparum*; PQ, primaquine; G6PD, Glucose-6-phosphate dehydrogenase; G6PDd, G6PD deficient; FST, fluorescent spot test; DOT, directly observed treatment; SLD, single low dose (0.25 mg/kg); CQ, chloroquine; ND, no data.

* Prevalences shown from reference [24] correspond to modelled national-level allele frequencies; other estimates shown are mostly from G6PD deficiency quantitative surveys.

** Policies from the WHO World Malaria Report (2016) were updated with the WHO 2017 report after this paper was reviewed [28]. Primaquine is currently contraindicated in infants and pregnant and breastfeeding women; ‘single dose’ refers to a dose of either 0.25 mg/kg or 0.75 mg/kg, and SLD refers to 0.25 mg/kg dose only. The predominant G6PD variant across the African region is G6PD A-; however, there are occasional reports of other variants, e.g., Mediterranean (South Africa, Sudan, Comoros) and Santamaria (Senegal, The Gambia) [24].

° Policies from the WHO 2016 report were checked with sources in these countries; *corresponding updates are shown in italics*.

Cells shaded in grey indicate countries are in the elimination phase.

**Fig 1:**
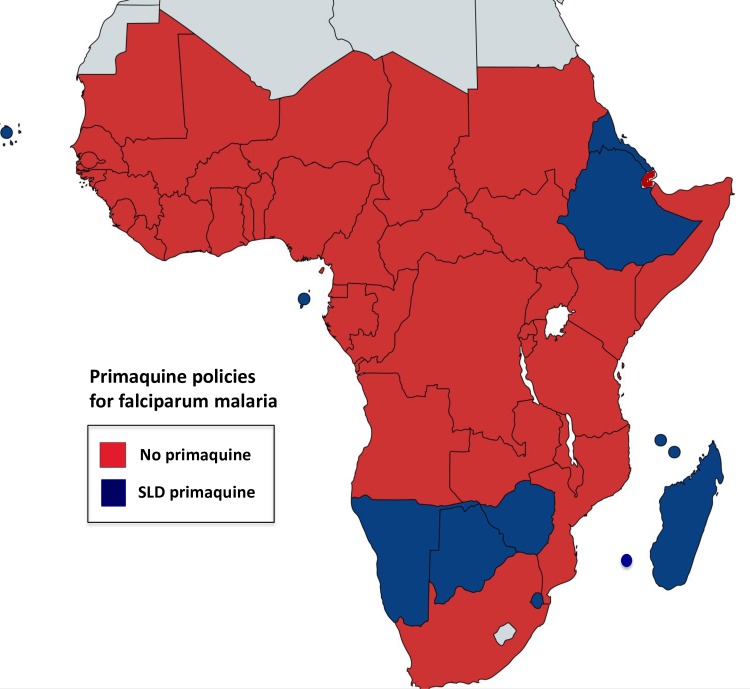
Single-dose primaquine policies in African countries. Most countries do not include primaquine in their malaria treatment policies (shown in red). Countries that use single low dose primaquine (SLD of 0.25 mg/kg, for most countries only very recently adopted) as policy for falciparum treatment are shown in dark blue. For specific details on countries’ policies and implementation and/or upcoming modifications, refer to Table 4. The map was created using an online tool [87].

**Fig 2:**
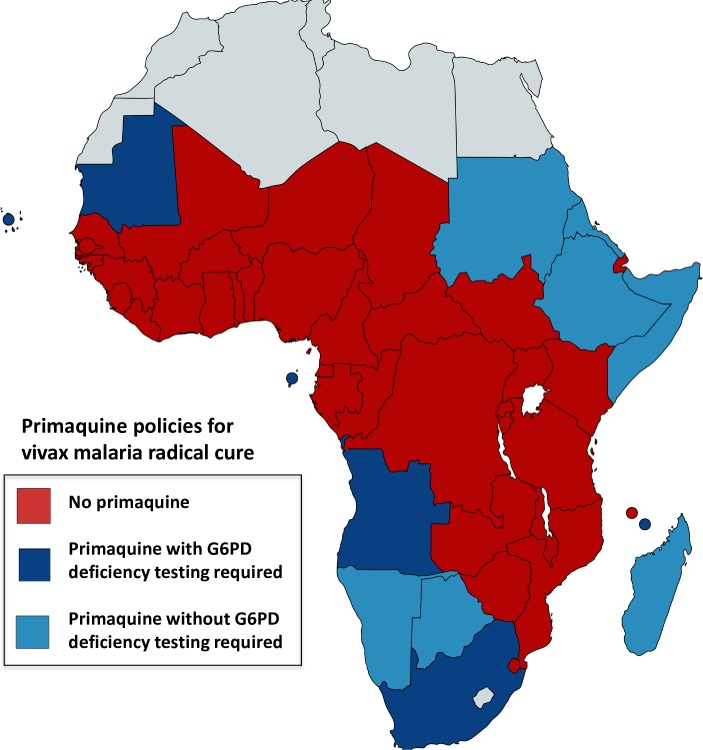
Radical cure primaquine policies in African countries. Most countries do not include primaquine in their malaria treatment policies or have no policy (shown in red). Countries that have radical cure for vivax malaria (0.25 mg/kg/day for 14 days) as policy are shown in blue; dark blue: G6PD deficiency testing required prior to primaquine treatment, light blue: testing not required. Table 4 provides specific details on countries’ policies and implementation and/or upcoming modifications.

Most countries are in the malaria control phase, except for Algeria, Botswana, South Africa, Cabo Verde, and Swaziland (now in elimination phase), and policy is not to use primaquine as a *P*. *falciparum* gametocytocide. The exceptions are Ethiopia, where single low dose (SLD 0.25 mg/kg) is now accepted although not yet widely implemented, and Madagascar, Botswana, Eritrea, Swaziland, Zimbabwe, Mauritania, which recently included single-dose primaquine as policy (2015), and Comoros, Mayotte, Namibia, Sao Tome and Principe, Algeria, and Cabo Verde (Table 4 and Fig 1). Primaquine is seldom available for radical cure of vivax or ovale malaria. In terms of official policy, except for Angola, Cabo Verde, Mauritania, South Africa, Mayotte, Sao Tome and Principe, and Equatorial Guinea, most countries either do not require G6PD deficiency testing before primaquine administration for radical cure or do not have a G6PD deficiency testing policy. Ethiopia is planning to use primaquine for *P*. *vivax* in districts targeted for malaria elimination, together with G6PD deficiency testing. Madagascar, Botswana, Cabo Verde, Mayotte, Sao Tomé and Principe, Namibia, and Mauritania have modified their policies recently (2014–2015) to include primaquine for radical cure (0.25 mg/kg for 14 days), but Botswana, Namibia, and Madagascar do not require G6PD deficiency testing before primaquine, while Mauritania, Mayotte, Sao Tomé and Principe, and Cabo Verde do (Table 4 and Fig 2).

### Middle East, Asia, and Oceania

The enormous populations of this large region host a heterogeneous array of G6PD variants [30]. While the prevalence of G6PD deficiency in malaria-endemic countries is generally lower than in Africa, it exceeds 13%–17% in 4 countries and is around >10%–13% in 2 others (Table 5). Most of the variants are at the more severe end of the polymorphic spectrum. Several common Asian variants were categorised as severely deficient (1%–10% residual activity) in the original WHO Working Group severity classification. These include Viangchan, Coimbra, Kaiping, Union, Mediterranean, Valladolid, and Gaohe [24,31–33]. The severe G6PD Mediterranean variant predominates over much of Europe, the Middle East, and West and Central Asia. The Mahidol variant, which is common in Myanmar and adjacent countries, was classed as moderate (Class III; 10%–60% residual activity), but recent studies indicate severity similar to the G6PD Viangchan variant (common in Lao PDR and Eastern Thailand) [34]. Although typical Asian variants would be expected to have a high haemolytic anaemia risk during radical cure treatment with primaquine, apart from the A-, Mahidol, Viangchan, and Mediterranean variants, there is very limited quantitative information on haemolysis in the different G6PD deficiency variants.

**Table 5:** G6PDd prevalence, testing, and primaquine policies in Asian, Oceania, and Middle East malaria-endemic countries.

Country	Estimated G6PD deficiency prevalence for common variants*	G6PD variants [24]	G6PD deficiency testing before primaquine **	Single-dose primaquine for Pf**	Primaquine use for Pv**
Thailand°	>13%–17% [24]	Mahidol, Viangchan, Canton, Kaiping, Mediterranean, Songklanagarind, Union, Vanua Lava, Chinese‐5, Gaohe, Kerala‐Kalyan, Quing Yan	Yes (adopted 2015) *but with poor implementation*, *PQ administered without testing*	Single dose without G6PD deficiency testing as DOT	In G6PDd 0.25 mg/kg daily for 14 days as DOT
Malaysia°	>7%–10% [24]	Andalus, Canton, Chatham, Coimbra, Kaiping, Mediterranean, Namouru, Nankang, Union, Vanua Lava, Viangchan‐Jammu, Chinese‐5, Gaohe, Mahidol, Orissa, Quing Yan [24]	Required since 1993, *done routinely*	Single dose since 2013; PQ given as DOT	ACT and PQ at 0.5 mg/kg/day for 14 days as DOT; *in practice*, *PQ not given as DOT and only given to non-G6PDd individuals*
Myanmar°	>3%–7% [24]	G6PD Mahidol, Canton, Coimbra, Viangchan‐Jammu, Kaiping, Mediterranean, Union, Valladolid, Kerala‐Kalyan [24]	No	*Single dose 0*.*75 mg/kg;* PQ given as DOT since 2014	CQ and PQ at 0.5 mg/kg/day for 14 days *at health centres and 0*.*75 mg/kg weekly dose for 8 weeks given by malaria volunteers in the community;* PQ given as DOT since 2014
Lao People’s Democratic Republic°	>13%–17% [24]	G6PD Viangchan [24]	Required since 2010 *but not available at village level*	*SLD (0*.*25 mg/kg)*	*0*.*25 mg/kg/day for 14 days in G6PD-normal patients*, *0*.*75 mg/kg/week for 8 weeks for G6PDd*
Cambodia°	>13%–17% [24] In Pailin, western Cambodia, *N* = 938 tested for G6PDd by FST and RDT for 74 samples (7.9%) moderate and severe G6PD deficiency (activity <30%), mostly in male population [62]	G6PD Viangchan, Kaiping, Canton, Valladolid, Mahidol, A- [24]	Required since 2012 Currently, there is no G6PD testing in the field. CareStart test will be piloted for field use at health centre level	Yes since 2014 but not implemented; *plan to implement SLD 0*.*25 mg/kg on first day without G6PD deficiency test*	Yes since 2013 but not implemented; *upcoming implementation planned (0*.*75 mg/kg weekly for 8 weeks or 0*.*25 mg/kg daily for 14 days*, *depending on predicted patient’s adherence)*
Vietnam°	>7%–10% [24] G6PD Viangchan Male outpatients, hospital in southern Vietnam *N* = 1,104 tested: 25 G6PDd (2.3% prevalence) Genotyping: novel mutation (352T>C or 118Tyr>His, ‘Bao Loc’) with activity <10%; Viangchan commonest (6/19), followed by Canton (5/19), Kaiping (3/19), Gaohe (1/19), Quing Yuan (1/19), and Union (2/19) [63]	Viangchan, Canton, Kaiping, Union, Bao Loc, Coimbra, Chinese‐5, Gaohe, Mahidol, Quing Yan [63]	Not required	Single dose since 2003 on last day of treatment as DOT	CQ and PQ at 0.25 mg/kg/day for 14 days since 2013 as DOT; *due to low PQ availability*, *not many patients take the full dose*
Bangladesh°	>3%–7% [24] Patients with confirmed malaria infection (*N* = 174) tested by spectrophotometry: G6PD deficiency prevalence 3.45%: 1 severe deficiency (<10% activity), 5 mild deficiency [64], 0.7% prevalence by FST in 141 Bengali falciparum malaria patients in southern Bangladesh [65] 6% severe deficiency in Chittagong Hill Districts populations (Marma and Khyang ethnic groups) [65]	Orissa, Kerala-Kalyan, and Mahidol [65,66]	Not required; *PQ given with advice to check for side effects of haemolysis; if so*, *stop PQ and refer the patient to hospital*	Single dose *0*.*75 mg/kg on the first day of treatment*	CQ and PQ at 0.25 mg/kg/day for 14 days since 2004
Nepal°	>3%–7% [24]	Mediterranean [24]	Required *but not widely available*	Single dose 0.25 mg/kg since 2015 but not implemented. *SLD 0*.*25 mg/kg on the first day of treatment*	0.25 mg/kg daily for 14 days since 2004
India°	>7%–10% [24] Tribal groups (*N* = 72 tribes) from 56 districts (data collected by field surveys using different methods) showed G6PD deficiency varying from 2.3%–27%; overall prevalence 7.7% [67]	Mediterranean (most common), Kerala-Kalyan, Odisha [24], Chatham, Coimbra, Namouru, Nilgiri, Orissa [24]	Not required; *done in urban areas (approximately 10% patients get tested)*	*PQ use without G6PD deficiency testing at single dose 0*.*75 mg/kg on day 2 with ACT*	0.25 mg/kg daily for 14 days as DOT since 2007; although *DOT not really implemented*, *health workers get paid for ensuring compliance*. *Patients are instructed to stop treatment and see a health worker if dark urine or bluish lips occur*
Pakistan°	>13%–17% [24]	Mediterranean, Chatham, Orissa [24]	Required since 2009, *although rarely available; also lack of consensus regarding risks and benefits*	Single dose since 2012	CQ and PQ at 0.25 mg/kg/day for 14 days
Afghanistan°	7%–10% [24] High prevalence of G6PD Mediterranean, overall 5.6%; in the Pashtun/Pashai group 8.9%, compared to 2% in the rest of the population [68]	G6PD Mediterranean [24]	Required since 2010; *however*, *there is very low availability of G6PD deficiency tests*	Single dose DOT since 2014; however, PQ *may be given to take at home on day 3*	CQ and PQ as DOT at 0.75 mg/kg weekly for 8 weeks; *Pv weekly regimen mostly unobserved*
Yemen	5%–6% in male population [69]	No published G6PD types	Required	Single dose implemented recently	CQ and PQ at 0.25 mg/kg/day for 14 days
Iran°	>10%–13% [24]	G6PD Mediterranean [70] and Chatham [71] and Cosenza [72]	Not required	Use without G6PD deficiency testing as single dose as DOT *on day 3*	0.75 mg/kg weekly for 8 weeks as DOT
Saudi Arabia	>10%–13% [24]	G6PD Mediterranean (most common), G6PD-Med-like, Aures, Chatham, Kaiping, S. Antioco, Union, Viangchan [24]	Required since 1985	Single dose	CQ and PQ at 0.25 mg/kg/day for 14 days
China°	>3%–7% [24] In southern China (Jiangxi province) among Chinese Hakka (*N* = 2,331) screened by a fluorescent test, 3.60% G6PD deficiency prevalence was found [73]. In Chaozhou region of eastern Guangdong Province, 3.36% (142/4224) G6PDd overall—2.33% (47/2013) males and 4.3% (95/2208) females [74]	Kaiping, Canton, Gaohe, Chinese-5, and Quing Yan Chinese‐1, Coimbra, Fushan, Haikou, Hechi, Liuzhou, Miaoli, Nankang, Songklanagarind, Taipei, Taipei‐Hakka, Union, Valladolid, Viangchan, A‐, Guangzhou, Keelung, Mahidol, Nanning, Quing Yan, Ube Konan [24,75,76]	Not required *in central China*, *where G6PD deficiency is very low (2–5 per million); only in Yunnan and Hainan provinces (G6PDd 1%–10%*, *PQ not used before 2010) there will be G6PD deficiency testing for Pv cases*	Single dose as DOT since 2013; *however*, *not implemented due to the absence of Pf local transmission*	Without G6PD deficiency testing as DOT at 0.75 mg/kg for 8 days *in central China*, *and in Yunnan and Hainan provinces*, *G6PDd cases will be treated with 0*.*75 mg/kg weekly for 8 weeks*
Bhutan	>3%–7% [24]	None published	Not required	Single dose since 2012	CQ and PQ at 0.25 mg/kg/day for 14 days *without observation* [77]
Indonesia°	>7%–10% in Indonesia [24] *N* = 2,033 residents of 3 separate districts in western Sumba (eastern Indonesia); 104 (5.1%) G6PDd by activity measured by commercial kit [77]. In western Sumba Island by quantitative assay, 7.2% (44/610) G6PDd overall, 9.2% males (24/260), 5.7% (20/350) females [78]	Vanua Lava, Viangchan, Chatham, Canton, Coimbra, Kaiping, Mediterranean, Surabaya, Union, Bajo Maumere, Chinese‐5, Gaohe, Mahidol [77]	Not required	Single dose *0*.*75 mg/kg is implemented widely; policy will change to the SLD 0*.*25 mg/kg in 2017*	0.25 mg/kg/day for 14 days with ACT
Timor-Leste	No published estimates	None published	Required since 2016	No policy	CQ and PQ at 0.75 mg/kg once weekly for 8 weeks as DOT
Philippines°	>1%–3% [24]	Union [24]	Required since 2009—*poor implementation*, *PQ administered without testing*	Single dose since 2006; PQ given as DOT since 2010	CQ and PQ at 0.5 mg/kg/day for 14 days *(but usually given at 0*.*25 mg/kg/day*); as DOT since 2010
Papua New Guinea°	>7%–10% [24]	G6PD Viangchan [24]	Not required	Not used, *guidelines are being updated to include SLD 0*.*25 mg/kg given on first day of treatment*	0.25 mg/kg/day for 14 days with ACT
Vanuatu	6.8% in males [79]	Namouru, Naone, Union, Vanua Lava [24]	Required	No policy	CQ and PQ at 0.25 mg/kg/day for 14 days
Solomon Islands	15.7%–30.9%[24]	Union [24]	Required	No policy	CQ and PQ at 0.25 mg/kg/day for 14 days
Republic of Korea	>0%–1% [24] In a vivax malaria endemic region, from *N* = 1,044 tested quantitatively, none were G6PDd [80]	Sporadic reports of uncommon variants [81]	Not required	No policy—there is no *Pf* malaria in the country	CQ and PQ at 0.25 mg/kg/day for 14 days
Democratic People’s Republic of Korea	>0%–1% [24]	None published	Not required	No policy—there is no *Pf* malaria in the country	CQ and PQ at 0.25 mg/kg/day for 14 days as DOT

**Abbreviations:** *Pv*, *Plasmodium vivax*; *Pf*, *Plasmodium falciparum*; G6PD, Glucose-6-phosphate dehydrogenase; G6PDd, G6PD deficient; CQ, chloroquine; PQ, primaquine; DOT, directly observed treatment; RDT, rapid diagnostic test; FST, fluorescent spot test; SLD, single low dose (0.25 mg/kg); ACT, artemisinin combination therapy.

*Prevalences shown from reference [24] correspond to national-level allele frequencies modelled as described therein; other estimates shown are mostly from G6PD quantitative surveys.

**Policies from WHO World Malaria Report 2016 were updated with the WHO 2017 report after this paper was reviewed [28]. Primaquine is currently contraindicated in young infants and pregnant and breastfeeding women; ‘single dose’ refers to a dose that may be 0.25 mg/kg or 0.75 mg/kg.

° Policies from the WHO 2016 report were checked with these countries; *corresponding updates are shown in italics*.

Cells shaded in grey indicate countries are in the elimination phase.

### Primaquine policies

**Fig 3:**
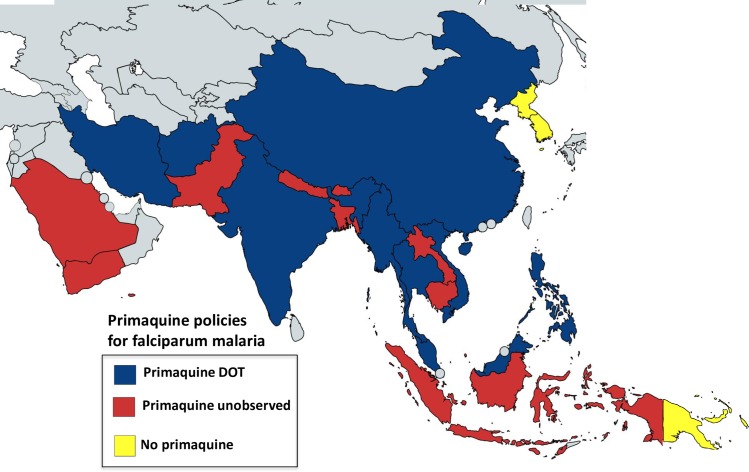
Single-dose primaquine policies in Asia, Oceania, and Middle East countries. All countries except for Papua New Guinea and both Koreas (which have no falciparum malaria) have policies to use primaquine as single dose for falciparum malaria. Red indicates unobserved therapy policy, and dark blue directly observed therapy (DOT). Implementation of primaquine policies and exact dose (SLD of 0.25 mg/kg or 0.75 mg/kg) vary between countries. Table 5 provides details on countries’ policies implementation and/or upcoming modifications.

Most countries in Asia have low seasonal transmission, and in recent years, many have set ambitious targets for malaria elimination within the next decade. The emergence and recent spread of artemisinin-resistant *P*. *falciparum*, leading to ACT failure in 5 countries of the Greater Mekong subregion (Cambodia, Lao People’s Democratic Republic, Myanmar, Thailand, and Vietnam) [35], threatens these targets. In this context, addition of SLD primaquine to falciparum malaria treatment regimens has been encouraged to reduce transmissibility of the treated infection. Several countries were already recommending the higher single gametocytocidal primaquine dose (0.75 mg/kg) without concerns. Except Papua New Guinea, Vanuatu, Solomon Islands, and Timor-Leste, all countries now recommend adding a single dose of primaquine to ACTs for falciparum malaria (Fig 3 and Table 5). Cambodia has just adopted this policy (SLD 0.25 mg/kg), and Papua New Guinea is updating its policy.

**Fig 4:**
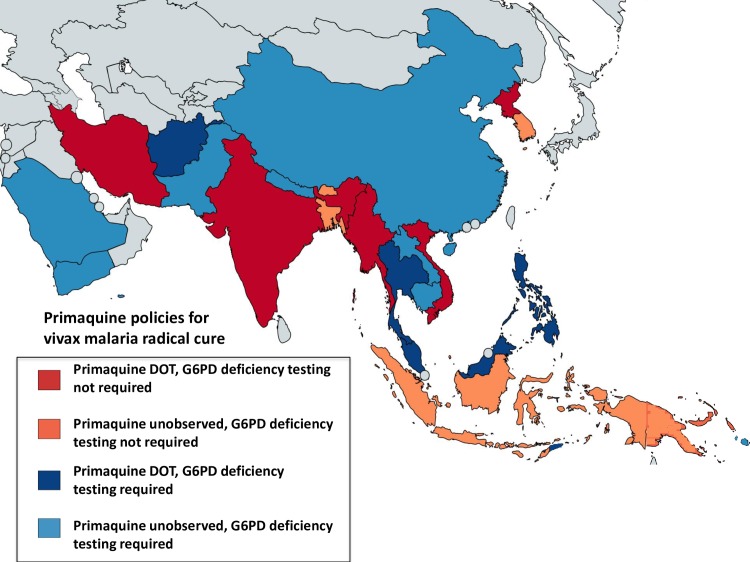
Radical cure primaquine policies in Asia, Oceania, and Middle East countries. All countries have policies to use primaquine for radical cure of vivax malaria, mainly as 0.25 mg/kg/day for 14 days, with some countries using the weekly regimen (0.75 mg/kg once weekly for 8 weeks). Colours indicate requirement for G6PD deficiency testing before primaquine administration: blue—G6PD deficiency testing required, red—testing not required, with light red or blue for unobserved therapy and dark red or blue for directly observed therapy (DOT). Implementation of these policies, regimens used, and requirement for G6PD deficiency testing vary between countries. Table 5 provides details on countries’ policies, implementation, and/or upcoming modifications.

Radical cure primaquine treatment policies vary across the Asian region. Some countries state a requirement for G6PD deficiency testing before primaquine administration (Lao People’s Democratic Republic, Cambodia, Pakistan, Afghanistan, Malaysia, Saudi Arabia, Sri Lanka, Philippines, Thailand, Yemen, Timor-Leste, Vanuatu, and Solomon Islands), although in practice testing is often not available. Myanmar acknowledges the lack of access to testing at village level, where most malaria cases are diagnosed, and has recently adopted a policy of weekly primaquine administration for 8 weeks for all patients treated by community malaria volunteers. Iran also recommends this regimen (Table 5). While it is policy to administer the radical cure primaquine regimen as DOT in the Democratic People’s Republic of Korea, Malaysia, Iran, Sri Lanka, Philippines, India, Thailand, Vietnam, Timor-Leste, and Afghanistan (Fig 4), this has not been implemented widely in the latter 3 countries. A survey of antimalarial availability and use among physicians, pharmacists, and patients across 6 states in India in 2008 found primaquine was prescribed for confirmed vivax malaria in 87% of patients in the public sector and 52% in the private sector [36]. In most other countries, very little primaquine is prescribed from the private sector, yet in many countries, this is the main source of antimalarials (Lao PDR [37], Cambodia, and Myanmar [38]).

Although WHO has recommended the higher daily dose of primaquine (0.5 mg/kg/day) for radical cure of *P*. *vivax* in East Asia and Oceania, most countries there still recommend the lower 0.25 mg/kg/day dose except for Malaysia, Myanmar, and the Philippines (Table 5).

Sri Lanka is currently in the prevention-of-reintroduction phase after having successfully eliminated malaria (certified by WHO as a malaria-free country in September 2016) [39]. China, Bhutan, Malaysia, the Republic of Korea, the Democratic People’s Republic of Korea, Saudi Arabia, and Iran are currently in the malaria elimination phase.

### The Americas

**Fig 5:**
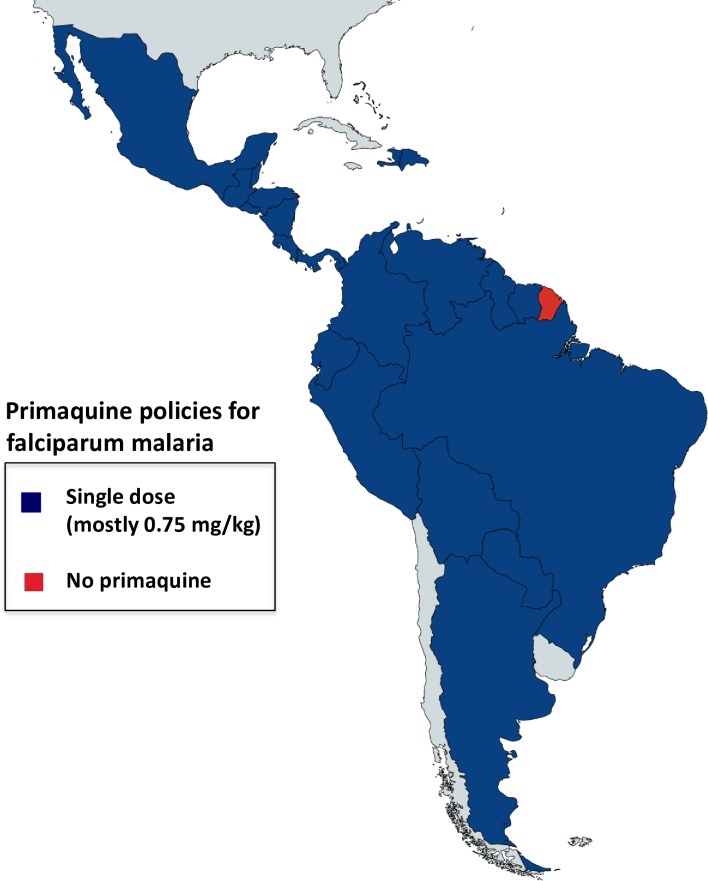
Single dose primaquine policies in American countries. All countries use single dose primaquine (mostly 0.75 mg/kg) as policy for falciparum treatment (shown in blue) except for French Guiana (red), with no G6PD deficiency testing required before primaquine administration. Table 6 provides details on countries’ policies and implementation and/or upcoming modifications.

Several countries in Central and South America are well on the path towards malaria elimination (Mexico, Belize, Costa Rica, Dominican Republic, Ecuador, El Salvador). Paraguay has applied to be certified as malaria-free, and Argentina is in the prevention-of-reintroduction phase. In sad contrast, malaria has resurged in Venezuela as the public health services there have weakened. The majority of malaria cases in the Americas come from the Amazon region [40]. There is heterogeneity of G6PD variants [30,41], with a predominance of A- [41]. There are limited data on the distribution of G6PD deficiency variants in areas where malaria is endemic. With the exception of French Guiana, all countries currently recommend primaquine as a *P*. *falciparum* gametocytocide (Table 6 and Fig 5), mostly at 0.75 mg/kg. This will be lowered to 0.25 mg/kg in Brazil’s revised guidelines, and Colombia has just updated the national malaria treatment guidelines to include primaquine single dose.

**Table 6:** G6PD deficiency prevalence, testing, and primaquine policies in American malaria-endemic countries.

Country	Estimated G6PD deficiency prevalence for common variants*	Is primaquine given as DOT?**	Single-dose primaquine for Pf**	Primaquine use for Pv**
Brazil°	>3%–7% [24] 0%–12.9% [40] G6PD A-, Mediterranean, Santamaria, Amazonia, Ananindeau, Belem, Crispim, Chatham, Farroupilha, Bahia, Lages, Seattle, Seattle‐like	No	Single dose *on first day to change soon from 0*.*75 mg/kg to SLD 0*.*25 mg/kg*	CQ and PQ at 0.50 mg/kg/day for 7 days; *upcoming guidelines will include a recommendation for G6PD deficiency testing*, *for a Pv diagnosis*, *before PQ; G6PDd cases to be treated with PQ at 0*.*75 mg/kg/week for 8 weeks*
Colombia°	>3%–7% [24] 1.4%–15.4% [40] 6.56%, only A and A- variants found [82,83]	No	PQ 0.75 mg/kg, single dose *(guidelines updated recently)*	CQ and PQ at 0.25 mg/kg/day for 14 days
Peru°	>0%–1% [24] 0%–0.7% [40]	Yes	*PQ 0.75 mg/kg single dose on the last day of ACT, since 2015 [84]*	CQ and PQ at 0.50 mg/kg/day for 7 days
Venezuela	>7%–10% [24] 0%–3.5% [40] In eastern areas (*N* = 664), a quantitative method showed overall 24 (3.6%) G6PD deficiency (4.5 ± 1.2 U/g Hb activity), with A- (202A/376G) variant in 17 (70.8%) individuals: 13 males, 4 heterozygous females [84]	No	Single dose	CQ and PQ at 0.25 mg/kg/day for 14 days
Bolivia	>0%–1% [24] 0.7%–1.5% [40]	Yes	Single dose but not implemented	CQ and PQ at 0.5 mg/kg/day for 7 days since 2001
Panama°	>0%–1% [24] G6PD A- and Mediterranean	Yes	Single dose on the first day with AL, since 2012	CQ and PQ at 0.25 mg/kg/day for 14 days
Guatemala°	>1%–3% [24]	Yes	Used but *not as a single dose; instead given daily for 3 days (15 mg each for 45 mg total or 0*.*75 mg/kg)*	CQ and PQ at 0.25 mg/kg/day for 14 days
Nicaragua	>1%–3% [24]	Yes	Single dose on the first day of CQ, since 2013	CQ and PQ at 0.5 mg/kg/day for 7 days
Haiti°	>7%–10% [24]	*Pf single dose is given DOT at health centres*	Single dose *0*.*75 mg/kg* on the first day of CQ	CQ and PQ at 0.25 mg/kg/day for 14 days; *PQ contraindicated in G6PDd patients*
Honduras	>1%–3% [24] *N* = 398 archival DNA samples of malaria patients [86]. G6PD A- assessed by PCR–RFLP and a commercial kit. Overall frequency 16.08%; A- was 11.9% (4.1% males; 1.5% homozygous and 6.3% heterozygous females). One Santamaria [85]	No	0.75 mg/kg as single dose the first day of treatment	According to national guidelines, malaria treatment administration of both CQ and PQ at 0.25 mg/kg/day for 14 days
Suriname°	>0%–1% [24]	No	Single dose *0*.*75 mg/kg* with AL since 2004, *when possible on first day of treatment*	CQ and PQ at 0.25 mg/kg/day for 14 days since 2004; *considering to increase to 0*.*50 mg/kg/day 14 days*
French Guiana	>0%–1% [24]	No	Not used	With CQ, regimen not specified; G6PD deficiency test required
Ecuador	>3%–7% [24] 9.3%–12.8% [40]	No	Single dose with AL	CQ and PQ at 0.50 mg/kg/day for 7 days
Guyana°	>3%–7% [24]	No	Single dose on first day of treatment; *0*.*75 mg/kg single dose for Pf and recently updated guidelines include same treatment for P*. *malariae*	CQ and PQ at 0.25 mg/kg/day for 14 days
El Salvador, Paraguay°	>3%–7% [24] 2.4% El Salvador [40]	El Salvador only	Single dose on first day of treatment;	CQ and PQ at 0.25 mg/kg/day for 14 days
Mexico°, Belize°, Dominican Republic°	>1%–3% [24] 0%–12.7% Mexico [40] G6PD A-, Santamaria, Union, Valladolid, Vanua Lava, Viangchan, Mexico City, Seattle	Yes, except for Belize	Single dose on day 1; *Belize: 0.75 mg/kg for Pf on first day of CQ [87]; Dominican Republic: 0.75 mg/kg*	CQ and PQ at 0.25 mg/kg/day for 14 days; *Mexico: a 7-day course of CQ and PQ (0.5 mg/kg on all 7 days; Belize: either 14 (0.25 mg/kg) or 7 (0.5 mg/kg) days for Pv [87]; Dominican Republic: only has imported cases of Pv*
Argentina	>0%–1% [24] 0.3%–0.4% (males) [40]	Yes	Single dose	CQ and PQ at 0.25 mg/kg/day for 14 days
Costa Rica	>0%–1% [24] 0.4%–12.6% [39] G6PD A-, Santamaria	Yes	Single dose	CQ and PQ at 0.25 mg/kg/day for 14 days or 0.5 mg/kg/day for 7 days

**Abbreviations:** *Pv*, *Plasmodium vivax*; *Pf*, *Plasmodium* falciparum; CQ, chloroquine; PQ, primaquine; AL, artemether-lumefantrine; DOT, directly observed treatment; SLD, single low dose (0.25mg/kg); G6PD, Glucose-6-phosphate dehydrogenase; ACT, Artemisinin combination therapy; PCR-RFLP, polymerase chain reaction-restriction fragment length polymorphism.

*Prevalences shown from reference [24] correspond to national-level allele frequencies modelled as described therein; other estimates shown are mostly from G6PD quantitative surveys.

**Policies from WHO World Malaria Report 2016 [27] were updated with the WHO 2017 report after this paper was reviewed [28]. Primaquine is contraindicated in infants and pregnant and breastfeeding women; ‘single dose’ refers to a dose of either 0.25 mg/kg or 0.75 mg/kg, and SLD refers to 0.25 mg/kg dose only. G6PD deficiency testing not required before primaquine treatment in the Americas except in French Guiana.

° Policies from the WHO 2016 report were checked with these countries; *corresponding updates are shown in italics*.

Cells shaded in grey indicate countries are in the elimination phase.

**Fig 6:**
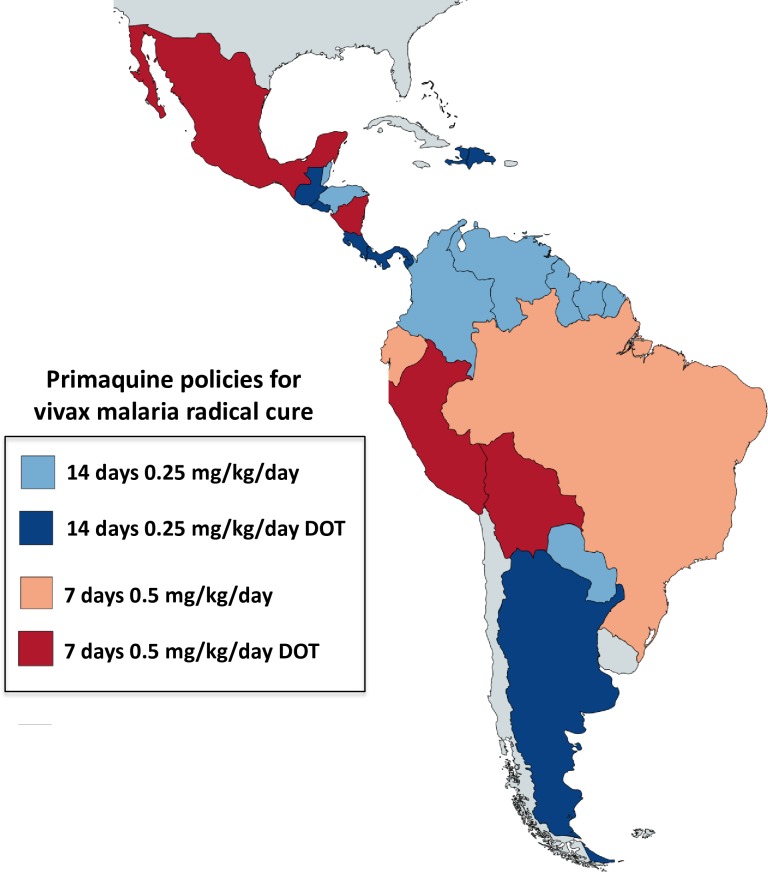
Radical cure primaquine policies in American countries. None of the countries requires G6PD deficiency testing before primaquine administration except French Guiana. Colours indicate the different regimens for radical cure of vivax malaria: blue—14 days, red—7 days, with light red or blue for unobserved therapy and dark red or blue for directly observed therapy (DOT). Table 6 provides specific details on countries’ policies and implementation and/or upcoming modifications.

*P*. *vivax* is estimated to have caused approximately 69% of the malaria cases in the Americas in 2015 [27,28]. None of the countries has as policy a requirement for G6PD deficiency testing before radical cure except French Guiana, although Brazil will include this recommendation in upcoming guidelines along with a recommended treatment of 0.75 mg/kg/week for 8 weeks for G6PDd individuals (Andre Siqueira, personal communication to Judith Recht, 2017). All countries recommend radical treatment of *P*. *vivax* malaria as policy. Primaquine 0.25 mg/kg for 14 days is given with chloroquine except in Brazil, Bolivia, Peru, Mexico, Nicaragua, and Ecuador, where the same total primaquine dose is recommended but given over 7 days (0.5 mg/kg/day for 7 days) (Table 6 and Fig 6). In Mexico, Dominican Republic, Nicaragua, Argentina, Peru, Bolivia, Costa Rica, Panama, Guatemala, and El Salvador, primaquine is administered as DOT.

## Discussion

There is substantial variation in national policies for the use of primaquine, even among countries with similar patterns of malaria epidemiology, drug resistance, and G6PD deficiency prevalence. This variation is amplified by the disconnect between policy and practice resulting from divergent opinions on risks and benefits. In many cases in this study, respondents confirmed discordance between the national recommendation and the current practice, invariably leading to primaquine underuse. For single-dose primaquine as a *P*. *falciparum* gametocytocide, the risk assessment is relatively easy, whilst the assessment of benefit is more difficult. The currently recommended primaquine SLD is 2–3 times lower than the 0.75 mg/kg dose recommended previously and 14–28 times lower than the total radical curative dose. In large studies of mass treatment conducted in areas where the G6PD Mahidol variant predominated, SLD primaquine was not associated with clinically significant haemolysis [42]. Given the information on the safety of multiple-dose radical cure including the implementation of weekly 0.75 mg/kg regimens in areas such as Iran, where the severe G6PD Mediterranean is prevalent, and the characterisation of the dose-response relationship for haemolysis in G6PDd individuals, it seems highly unlikely that SLD primaquine does pose a clinically significant haemolytic risk [21]. G6PD deficiency testing is, therefore, considered unnecessary in this case. It is unclear at what level of transmission intensity it is no longer cost beneficial to give single dose primaquine as a *P. falciparum* gametocytocide. However, as malaria transmission is reduced actively in an elimination programme, every transmission source must be removed. There is no clear explanation of why SLD primaquine is not deployed in all countries aiming for elimination. However, once the SLD primaquine policy is adopted, every effort should be made to ensure high coverage. Optimised age-based dosing regimens for SLD primaquine are recommended, based on anthropometric data for specific populations [43, 44]. An important impediment is the current lack of paediatric formulations [45, 46].

Use of primaquine in radical curative regimens carries much greater risks of haemolysis in G6PDd patients, but it also provides substantial benefits to treated patients [4]. The risks are systematically overestimated by surveys in healthy subjects as G6PD deficiency prevalences in malaria are usually lower because of the malaria protective effect, and unsurprisingly, this difference appears to be directly proportional to the degree of deficiency. While G6PD deficiency testing is recommended officially [47] in many countries, in reality, it is not available at point of care, leaving prescribers to make their own decisions. Apart from the Indian subcontinent, there is very little use of primaquine in the private sector [37,38], which in many countries is the main source of antimalarial treatment. Reported adherence to unobserved 14-day radical cure regimens varies between different studies (adherence was considered poor in Papua Indonesia [48], where most patients are children; in 2 studies in Thailand [49, 50]; and in studies from India, but good in other studies in Pakistan [an Afghan refugee camp] [51], Brazil [52], and the Thailand-Myanmar border [53]). This suggests that adherence is dependent on context and education practices. Although some countries recommend that primaquine be given as directly observed treatment (DOT) to allow the drug to be stopped if there are signs of haemolysis, the operational difficulties of providing a 14-day DOT may well mean many patients are partially treated or not given primaquine at all.

Where relapse rates are high (i.e., >50%, as in East Asia and Oceania), effective radical cure more than halves the incidence of vivax malaria. The benefit is substantial. If G6PD deficiency testing is unavailable, the correct approach to patient management is unclear as it depends on the prevalence and severity of G6PD deficiency in the patient’s original population, the degree of anaemia, and the availability of blood transfusion in case of severe haemolysis, balanced against the probability of relapse and its pathological consequences and the likely dose regimen required for radical cure. More could be done to improve the safety of primaquine administration through better packaging and presentation of the drug as for Coartem (artemether-lumefantrine) [54], which is marketed as age-targeted blister packs, with pictorial instructions on how it should be taken. Concern over haemolytic risk may explain why many countries in East Asia and Oceania recommend a daily dose of 0.25 mg/kg when there is good evidence for the radical cure superiority of the higher dose currently recommended by WHO [18]. For patients with *P*. *vivax* or *P*. *ovale* malaria who are G6PDd, WHO recommends they should not receive daily primaquine. Instead, these patients may receive once weekly primaquine 0.75 mg/kg for 8 weeks to prevent relapses, provided they are under close medical supervision for signs and symptoms of acute haemolytic anaemia during the first 3 weeks of treatment and they have access to health facilities with capacity for safe blood transfusion [55].

If vivax malaria is to be eliminated, use of radical curative treatment will have to increase. Some heterogeneity in policies for radical cure of vivax malaria is understandable, given the geographic variation in *P*. *vivax* relapse risks and the prevalence and severity of G6PD deficiency, but this review suggests there is plenty of room for improvement. The gap between policy and practice is also too wide, and primaquine is underused. Informed review of risks and benefits should help to harmonise treatment policies. As is often the case in malaria, the strength of opinion seems inversely proportional to the quality and quantity of the evidence, so more information is needed on the safety of radical cure regimens in the more severe G6PD deficiency variants to inform policies and practices. Measuring adherence accurately is very difficult, but as it is such a critical determinant of therapeutic response; it deserves further behavioural, pharmacokinetic, and epidemiological studies to try and ensure people do adhere to current and future regimens. Widescale availability of point-of-care G6PD deficiency testing would undoubtedly improve prescribing safety, although the current 30% activity threshold will not identify the majority of female heterozygotes. Safer primaquine regimens could be developed. A new 8-aminoquinoline tafenoquine will be introduced soon. This provides radical cure in a single dose, but as it should not be given to patients with less than 70% of normal G6PD activity, it will require deployment of a new G6PD deficiency test. This will still leave a significant minority of patients (particularly women of childbearing age) requiring a safe primaquine regimen. Policies and practices both need frequent review.

**Key learning points.**

- Glucose 6-phosphate dehydrogenase (G6PD) deficiency, the most common human enzymopathy, is prevalent in tropical and subtropical areas where malaria is or was endemic. Primaquine, the only drug licensed for radical cure of vivax and ovale malaria, and the only licenced antimalarial with specific gametocytocidal activity against *Plasmodium falciparum*, causes dose-dependent haemolysis in G6PD-deficient individuals.
- Primaquine is underused (reflected in both policies and practices) because of low availability of G6PD deficiency testing, poor adherence to radical cure regimens, lack of paediatric formulations, and concerns about toxicity.
- The single low dose of 0.25 mg/kg of primaquine recommended by WHO in 2012 to be added to falciparum malaria treatment to reduce transmission in low-transmission settings is safe but has not been adopted by many countries, particularly in Africa.
- A stronger commitment to implement primaquine treatment by malaria control programmes is needed. For current regimens recommended for radical cure of vivax and ovale malaria, this is unlikely to happen without expanded access to G6PD deficiency testing, patient education on toxicity warning signs, and improved dosage forms for children.

**Top five papers.**

1. Beutler E. The hemolytic effect of primaquine and related compounds: a review. Blood. 1959 Feb;14(2):103–39. PubMed PMID: 13618370.
2. Alving AS, Johnson CF, Tarlov AR, Brewer GJ, Kellermeyer RW, Carson PE. Mitigation of the haemolytic effect of primaquine and enhancement of its action against exoerythrocytic forms of the Chesson strain of *Piasmodium vivax* by intermittent regimens of drug administration: a preliminary report. Bull World Health Organ. 1960;22:621–31. PubMed PMID: 13793053; PubMed Central PMCID: PMC2555355.
3. Luzzatto L, Seneca E. G6PD deficiency: a classic example of pharmacogenetics with on-going clinical implications. Br J Haematol. 2014 Feb;164(4):469–80. doi: 10.1111/bjh.12665. Epub 2013 Dec 28. Review. PubMed PMID: 24372186; PubMed Central PMCID: PMC4153881
4. Howes RE, Piel FB, Patil AP, Nyangiri OA, Gething PW, Dewi M, Hogg MM, Battle KE, Padilla CD, Baird JK, Hay SI. G6PD deficiency prevalence and estimates of affected populations in malaria endemic countries: a geostatistical model-based map. PLoS Med. 2012;9(11):e1001339. doi: 10.1371/journal.pmed.1001339. Epub 2012 Nov 13. PubMed PMID: 23152723; PubMed Central PMCID: PMC3496665.
5. White NJ, Qiao LG, Qi G, Luzzatto L. Rationale for recommending a lower dose of primaquine as a *Plasmodium falciparum* gametocytocide in populations where G6PD deficiency is common. Malar J. 2012 Dec 14;11:418. doi: 10.1186/1475-2875-11-418. Review. PubMed PMID: 23237606; PubMed Central PMCID: PMC3546849.
